# Supplementary material for: Fermented Whey Ewe’s Milk-Based Fruit Smoothies: Bio-Recycling and Enrichment of Phenolic Compounds and Improvement of Protein Digestibility and Antioxidant Activity
Source: Antioxidants (Basel). 2023 May 12;12(5):1091. doi: 10.3390/antiox12051091 (PMC10215623; doi:10.3390/antiox12051091)

Figure S3. Separation by HPLC (245 nm) of ascorbic acid in extract obtained from fermented whey-fruit smoothie with *Lactiplantibacillus plantarum* BpL2 (BpL2_WFS) for 72 h at 30 °C. Peak assignments: 1, ascorbic acid.

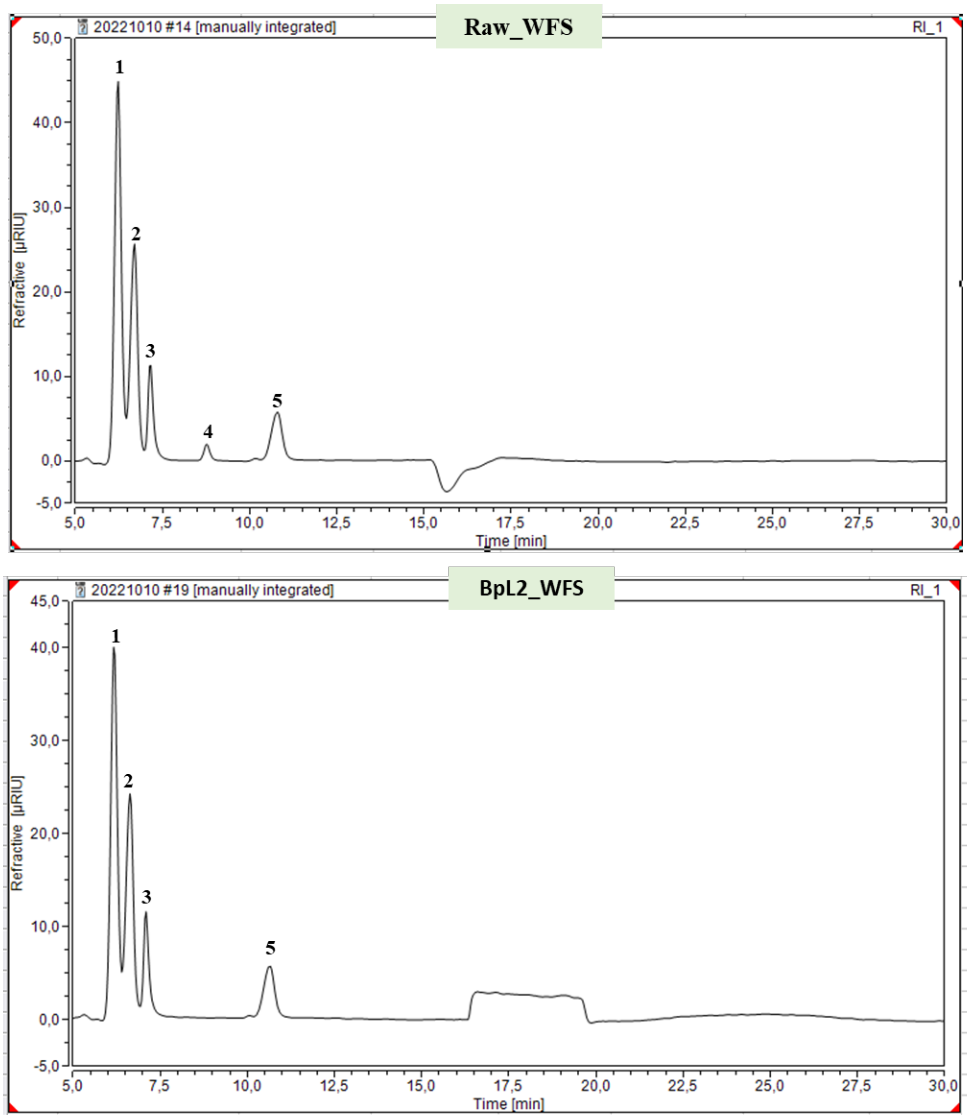

Supplement: Supplementary file 1 [file antioxidants-12-01091-s001.zip › Figure S3.pdf]
