# Supplementary material for: Fermented Whey Ewe’s Milk-Based Fruit Smoothies: Bio-Recycling and Enrichment of Phenolic Compounds and Improvement of Protein Digestibility and Antioxidant Activity
Source: Antioxidants (Basel). 2023 May 12;12(5):1091. doi: 10.3390/antiox12051091 (PMC10215623; doi:10.3390/antiox12051091)

Figure S4. Separation by by LC-ESI-MS/MS of phenolic compounds in methanol/water/ hydrochloric acid soluble extract (MWH-SE) obtained from fermented whey-fruit smoothie with *Lactiplantibacillus plantarum* BpL2 (BpL2_WFS) for 72 h at 30 °C.

Peak assignments: 1, gallic acid; 2, 3- hydroxybenzoic acid; 3, chlorogenic acid; 4, Hydrocaffeic acid; 5, procyanidin B2; 6, epicatechin; 7, vanillin; 8, *p*-coumaric acid; 9, ellagic acid; 10, isoquercetin; 11, phloridzin; 12, quercetin; 13, naringenin; 14, phloretin; and 15, isorhamnetin.

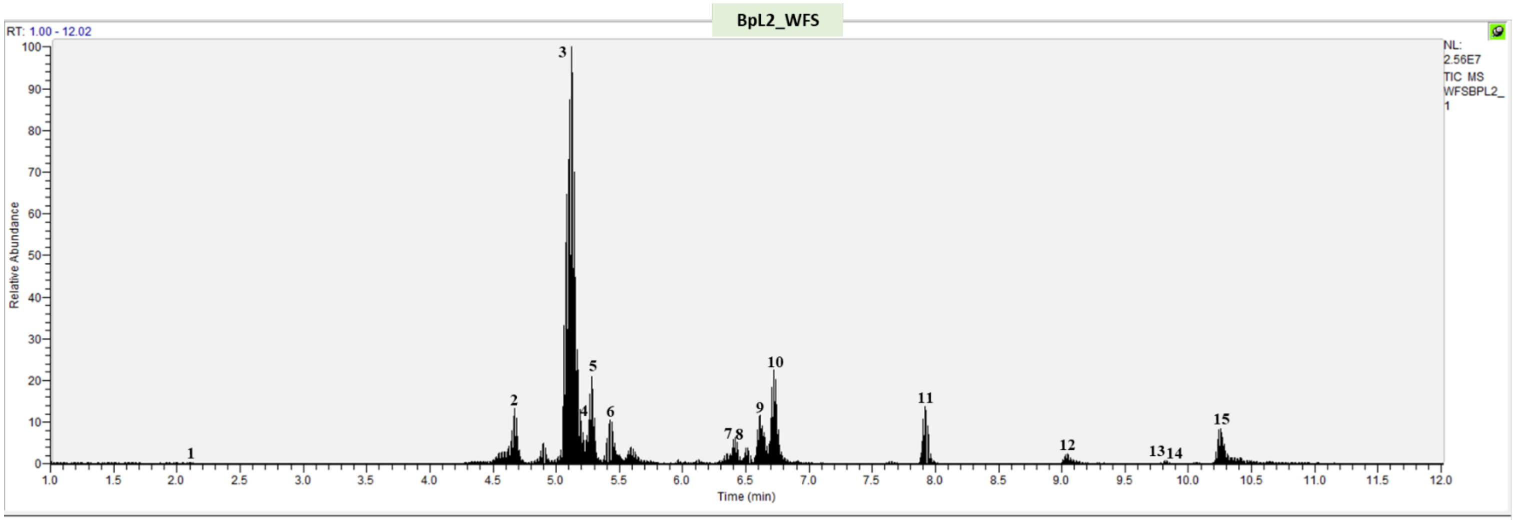

Supplement: Supplementary file 1 [file antioxidants-12-01091-s001.zip › Figure S4.pdf]
