# Supplementary material for: Fermented Whey Ewe’s Milk-Based Fruit Smoothies: Bio-Recycling and Enrichment of Phenolic Compounds and Improvement of Protein Digestibility and Antioxidant Activity
Source: Antioxidants (Basel). 2023 May 12;12(5):1091. doi: 10.3390/antiox12051091 (PMC10215623; doi:10.3390/antiox12051091)
Supplement: Supplementary file 1 [file antioxidants-12-01091-s001.zip › Table S1.pdf]

Table S1. List of lactic acid bacteria strains used in this study.

**Species**	**Code**	**Sources**
Leuconostoc mesenteroides	GL8	Apple
Lactiplantibacillus plantarum	GL9	Apple
L. plantarum	SL8	Strawberry
Leuc. mesenteroides	SL9	Strawberry
Leuconostoc holzapfelii	PHE5	Peach
L. plantarum	WSL1	Whey sheep
Lactococcus lactis	WSL2	Whey sheep
Leuc. mesenteroides	WSL3	Whey sheep
Leuconostoc pseudomesenteroides	WSL4	Whey sheep
L. plantarum	RPL3	Raspberry
L. plantarum	BPL2	Blueberry
Leuc. pseudomesenteroides	WGL2	Whey goat
Leuc. mesenteroides	WGL3	Whey goat
Leuc. mesenteroides	WCL1	Whey cow
Leuc. pseudomesenteroides	WCL4	Whey cow
Apilactobacillus kunkeei	BEE4	Honeybee
