# Supplementary material for: Fermented Whey Ewe’s Milk-Based Fruit Smoothies: Bio-Recycling and Enrichment of Phenolic Compounds and Improvement of Protein Digestibility and Antioxidant Activity
Source: Antioxidants (Basel). 2023 May 12;12(5):1091. doi: 10.3390/antiox12051091 (PMC10215623; doi:10.3390/antiox12051091)
Supplement: Supplementary file 1 [file antioxidants-12-01091-s001.zip › Table S2.pdf]

Table S2. Quantification of phenolic compounds (μg g^{-1} DW) by LC-ESI-MS/MS in methanol/water/hydrochloric acid soluble extract (MWH-SE) obtained from raw whey-fruit smoothie (Raw_WFS) and raw fruit smoothie (Raw_FS).

**Compounds**	**Raw_WFS**	**Raw_FS**
**Gallic acid**	0.31 ± 0.00[a]	0.23 ± 0.01[b]
**3- hydroxybenzoic acid**	13.32 ± 1.31	13.13 ± 4.15
**Chlorogenic acid**	62.94 ± 0.06[a]	45.05 ± 0.38[b]
***p*-coumaric acid**	9.39 ± 0.20	9.9 ± 0.20
**Phloridzin**	11.24 ± 3.24	11.5 ± 0.50
**Isorhamnetin**	5.6 ± 1.16	4.46 ± 0.01
**Naringenin**	1.46 ± 0.46	1.25 ± 0.04
**Phloretin**	6.15 ± 0.04	6.03 ± 0.00
**Quercetin**	0.93 ± 0.23	0.81 ± 0.01
**Isoquercetin**	3.59 ± 0.13[a]	1.47 ± 0.00[b]
**Epicatechin**	25.05 ± 0.5[a]	21.87 ± 0.22[b]
**Procyanidin B2**	25.46 ± 0.51	26.39 ± 0.2
**Ellagic acid**	8.7 ± 0.78[a]	6.01 ± 0.20[b]
**Vanillin**	6.14 ± 0.13	6.07 ± 0.03

[a–b] Means within the row with different letters are significantly different ($P < 0.05$).
