# Supplementary material for: Fermented Whey Ewe’s Milk-Based Fruit Smoothies: Bio-Recycling and Enrichment of Phenolic Compounds and Improvement of Protein Digestibility and Antioxidant Activity
Source: Antioxidants (Basel). 2023 May 12;12(5):1091. doi: 10.3390/antiox12051091 (PMC10215623; doi:10.3390/antiox12051091)
Supplement: Supplementary file 1 [file antioxidants-12-01091-s001.zip › Table S3.pdf]

Table S3. Quantification of anthocyanins compounds (µg g^{-1} DW) by LC-ESI-MS/MS in in methanol/water/trifluoracetic acid soluble extract (MWT-SE) obtained from raw whey-fruit smoothie (Raw_WFS) and raw fruit smoothie (Raw_FS).

Compounds	Raw_WFS	Raw_FS
**Cyanidin**	5.11 ± 1.65	4.12 ± 0.73
**Delphinidin**	11.52 ± 1.93	9.58 ± 0.00
**Malvidin**	6.3 ± 0.15[a]	5.22 ± 0.22[b]
**Peonidin**	2.97 ± 0.24	2.65 ± 0.03
**Petunidin 3-glucoside**	77.92 ± 15.08	106.3 ± 2.34

[a–b] Means within the row with different letters are significantly different ($P < 0.05$).
