# Supplementary material for: Fermented Whey Ewe’s Milk-Based Fruit Smoothies: Bio-Recycling and Enrichment of Phenolic Compounds and Improvement of Protein Digestibility and Antioxidant Activity
Source: Antioxidants (Basel). 2023 May 12;12(5):1091. doi: 10.3390/antiox12051091 (PMC10215623; doi:10.3390/antiox12051091)
Supplement: Supplementary file 1 [file antioxidants-12-01091-s001.zip › Table S4.pdf]

Table S4. Amino acids profile (mg g^{-1} DW) of raw whey milk-based fruit smoothie (Raw_WFS), WFS without microbial inoculum (Unstarted_WFS), and Started_WFS, which were incubated for 72 h at 30 °C. Fermentation (Started_WFS) was with selected single cultures of *Lactiplantibacillus plantarum* SL8 (SL8_WFS) and BpL2 (BpL2_WFS), *Leuconostoc holzapfelii* PHE5 (PHE5_WFS), *Lactococcus lactic* WSL2 (WSL2_WFS) and *Apilactobacillus kunkeei* BEE4 (BEE4_WFS).

	WFS_Raw	WFS_Unstarted	WFS_SL8	WFS_BpL2	WFS_PHE5	WFS_WSL2	WFS_BEE4
**Asp**	4.4	4.33	3.88	4.33	4.07	3.32	3.56
**Thr**	0.47	0.49	0.48	0.49	0.53	0.4	0.5
**Ser**	0.26	0.26	0.21	0.21	0.27	0.19	0.25
**Glu**	0.8	0.79	0.78	0.82	0.71	0.78	0.61
**Gly**	0.43	0.36	0.44	0.45	0.4	0.42	0.37
**Ala**	0.37	0.37	0.35	0.43	0.37	0.67	0.33
**Val**	0.15	0.14	0.13	0.15	0.12	0.12	0.12
**Cys +Met**	0.85	1.06	0.93	1.09	0.85	0.74	0.78
**Ile**	0.08	0.1	0.11	0.11	0.07	0.06	0.07
**Leu**	0.29	0.27	0.31	0.38	0.31	0.27	0.3
**Tyr+Phe**	0.48	0.4	0.43	0.58	0.49	0.57	0.52
**Lys**	0.32	0.31	0.28	0.26	0.33	0.36	0.37
**His**	0.15	0.15	0.13	0.17	0.15	0.13	0.13
**Trp**	0.49	0.49	0.5	0.52	0.53	0.51	0.46
**Arg**	0.07	0.09	0.12	0.12	0.1	0.09	0.08
**Pro**	0.66	0.65	0.64	0.78	0.63	0.55	0.59
**Total**	10.27	10.26	9.72	10.89	9.93	9.18	9.04
